# Crystal structure of methyl (4*R*)-4-(4-meth­oxy­benzo­yl)-4-{[(1*R*)-1-phenyl­eth­yl]carbamo­yl}butano­ate

**DOI:** 10.1107/S2056989017003607

**Published:** 2017-03-14

**Authors:** Alejandro Manchado, Mateo M. Salgado, Álvaro Vicente, David Díez, Francisca Sanz, Narciso M. Garrido

**Affiliations:** a Departamento de Química Orgánica, Universidad de Salamanca, Plaza de los Caidos, 37008-Salamanca, Spain; b Servicio de Difracción de Rayos X, Universidad de Salamanca, Plaza de los Caidos, 37008-Salamanca, Spain

**Keywords:** crystal structure, hydrogen bonds, CAN oxidation, β-lactame, glutarate

## Abstract

The title compound, C_22_H_25_NO_5_, was prepared by CAN [cerium(IV) ammonium nitrate] oxidation of the corresponding β-lactam. The dihedral angle between the benzene rings is 13.3 (4)° and the C—N—C(=O)—C torsion angle is 176.1 (6)°. In the crystal, amide-*C*(4) N—H⋯O and reinforcing C—H⋯O hydrogen bonds link the mol­ecules into infinite [010] chains. Further C—H⋯O hydrogen bonds cross-link the chains in the *c*-axis direction.

## Abstract 2

The CAN oxidation of a β-lactam leads to a 4-substituted glutarate. In the crystal, amide-*C*(4) N—H⋯O and reinforcing C—H⋯O hydrogen bonds link the mol­ecules into infinite [010] chains. Further C—H⋯O hydrogen bonds cross-link the chains in the *c*-axis direction.

## Chemical context   

Cerium(IV) ammonium nitrate (CAN) is a powerful reagent in organic synthesis, which promotes a wide range of reactions that go well beyond its usual role as an oxidant (Sridharan & Menendez, 2010 ▸). Chemoselective mono-de­benzyl­ation of benzyl tertiary amines occurs in the presence of *N*-benzyl amides, *O*-benzyl ethers and esters (Bull *et al.*, 2000 ▸); inter­estingly this reaction can be applied to mono-de­benzyl­ation of β-amino esters as a way to obtain β-lactams (Davies & Ichihara, 1998 ▸) or piperidone (Garrido *et al.*, 2011 ▸), providing as well a new oxidative methodology as catch linker for reaction monitoring and optimization on solid phase support (Davies *et al.*, 2008 ▸). Our group has demonstrated two different domino reactions, one by lithium amide addition to diendioate that can be applied to the synthesis of cyclo­penta­nic (Urones *et al.*, 2004 ▸) or cyclo­hexa­nic (Garrido *et al.*, 2006 ▸) derivatives and the other by addition to Baylis–Hillman (Garrido *et al.*, 2008 ▸) derivatives with application to the synthesis of non-peptidic neurokinin NK1 receptor antagonist (+)-L-733,060 (Garrido *et al.*, 2010 ▸). Within this context of the synthesis of biologically active compounds, we are inter­ested in the synthesis of *β*-lactam and its mono-deprotection, as shown in the Scheme[Chem scheme1], where the asymmetric 4-benzoyl glutarate is readily obtained by CAN oxidation of the appropriate substituted *β*-lactam. For the CAN oxidation reaction of a related trialkyl amine derivative providing monodeprotection, see Garrido *et al.* (2011 ▸).
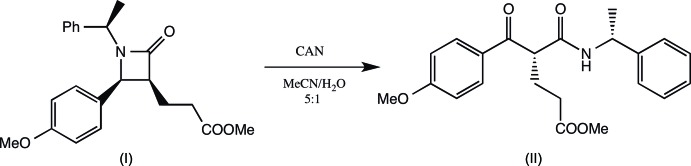


## Structural commentary   

**Figure 1:**
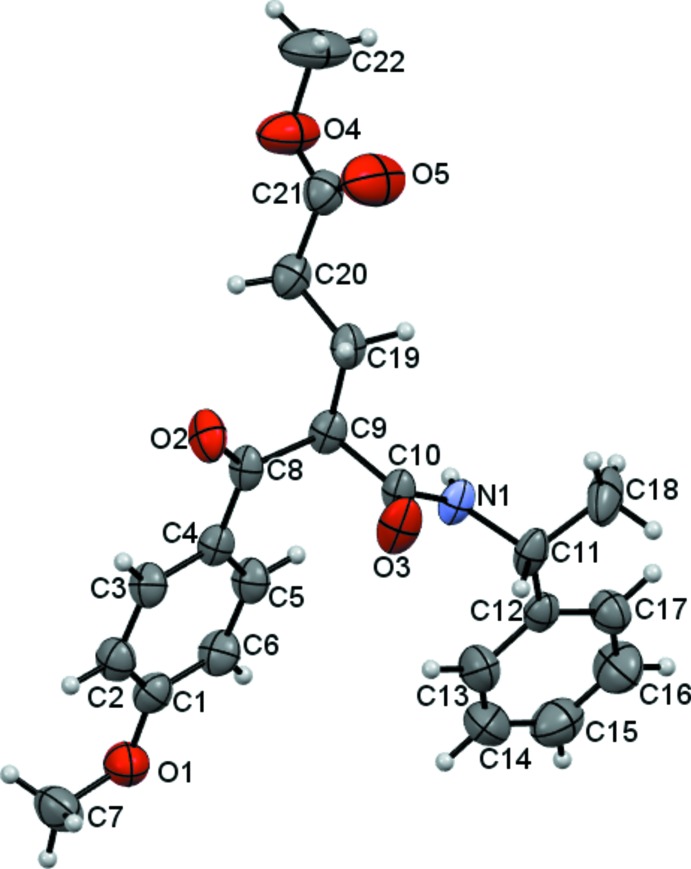
The mol­ecular structure of the title compound. Displacement ellipsoids are drawn at the 50% probability level. H atoms are shown as spheres of arbitrary radius.

The mol­ecular structure of the title compound is shown in Fig. 1 ▸. The mol­ecule consists of an ester amide glutarate derivative with a *p*-metoxybenzoyl group as substituent: all the bond lengths and angles are within normal ranges. The almost planar conformation of the ester group is established from the torsion angle C20—C21—O4—C22 of 178.6 (3)°. The ether group atom C1 and the carbonyl group atom C8 are almost coplanar with the benzene ring, the C7—O1—C1—C6 and O2—C8—C4—C5 torsion angles being 177.9 (1) and 172.4 (8)°, respectively. The C11 methyl group is also almost coplanar with the its benzene ring, as indicated by the torsion angle C18—C11—C12—C13 of 176.68 (7)°. The dihedral angle between the aromatic rings is 13.3 (4)°.

## Supra­molecular features   

**Figure 2:**
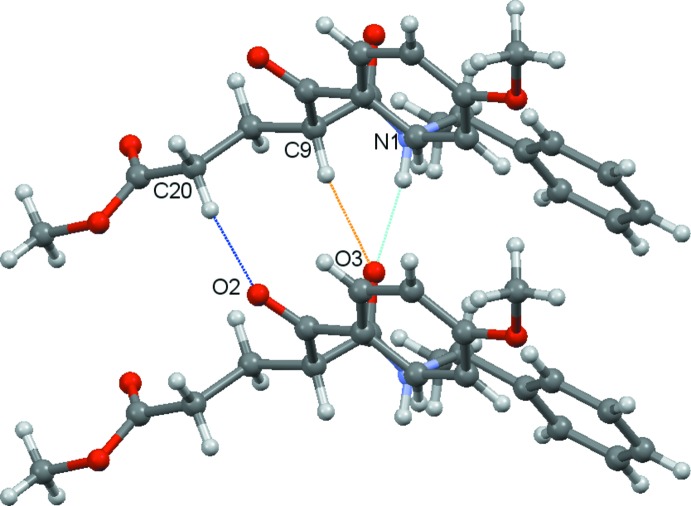
A view of the C20—H20*A*⋯O2 (dotted blue lines), N1—H1⋯O3 (dotted light-blue lines) and C9—H9⋯O3 (dotted orange lines) hydrogen bonds (see Table 1 ▸), which link the mol­ecules into [010] chains.

**Figure 3:**
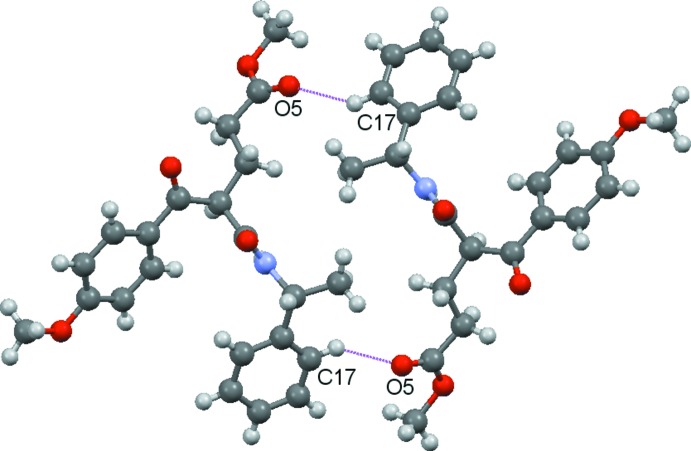
A view of the C17—H17⋯O5 (dotted pink lines) hydrogen bonds in the extended structure of the title compound.

**Figure 4:**
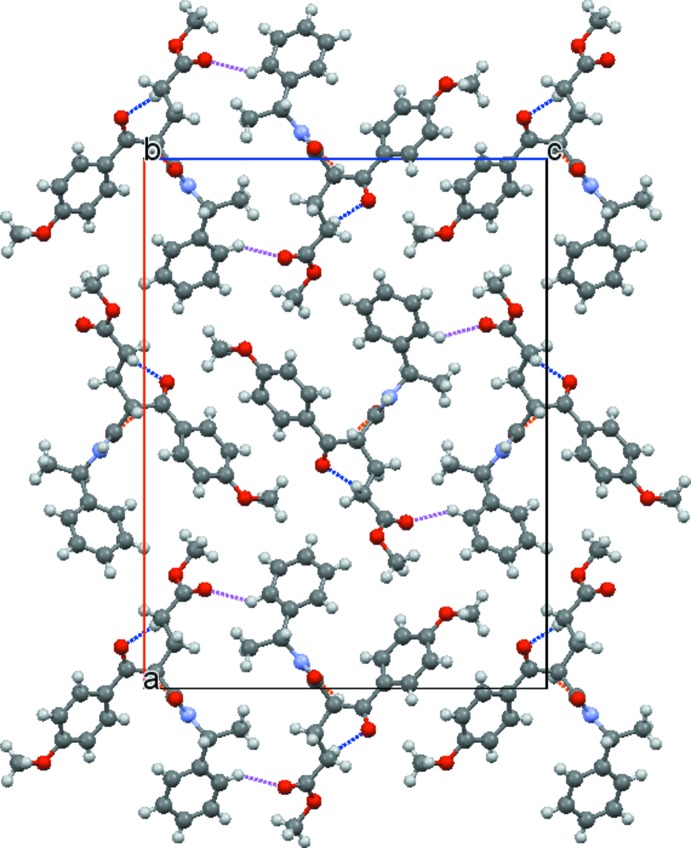
Crystal packing of the title compound, viewed along the [010] direction.

In the extended structure of the title compound, hydrogen bonds are one of the primary factors in building the crystal network (Table 1 ▸). Inter­molecular N1—H1⋯O3^i^ (dotted light-blue lines), C9—H9⋯O3^i^ dotted (orange lines) and C20—H20*A*⋯O2^i^ (dotted blue lines) hydrogen bonds link neighboring mol­ecules, generating infinite chains running along the *b-*axis direction (Fig. 2 ▸). These chains are joined to each other along *c* axis by C17—H17⋯O5^ii^ inter­actions (dotted pink lines), as shown in Fig. 3 ▸. The packing viewed along the [010] direction is illustrated in Fig. 4 ▸.

## Synthesis and crystallization   

(3*S*,4*S*,α*R*)-*N*-(α-methyl­benz­yl)-4-(*para*-meth­oxy­phen­yl)-3-meth­oxy­carbonyl­ethyl-β-lactam (I) (26.50 mg, 76.12 µmol) was dissolved in 12.00 ml of a mixture of MeCN–H_2_O (5:1) and CAN (150.1 mg, 0.27 mmol) was added and allowed to stir for 15 minutes under an argon atmosphere. Solid NaHCO_3_ was then added and the mixture allowed to stir for another 15 minutes. It was filtered over celite, washed with EtOAc and NaHSO_4_ and the phases separated. The organic phase was treated with H_2_O, brine and anhydrous Na_2_SO_4_, filtered and the solvent removed under reduced pressure to obtained the crude product (23.4 mg), which was purified by flash chromatography (silica gel, hexa­ne/EtOAC 7:3) and crystallized from hexa­ne/EtOAc solution to yield 7.7 mg of product (II) (28%), m.p. 440.6 K.

IR (film): 700, 802, 1026, 1171, 1260, 1373, 1456, 1512, 1601, 1736, 2849, 2918, 3333. ^1^H NMR (200 MHz, CDCl_3_) δ 8.33–7.78 (*m*, 2H, Ar), 7.48–7.25 (*m*, 5H, Ar), 6.99–6.81 (*m*, 2H, Ar), 5.05 (1H, *quint*, *J* = 6.9 Hz), 4.65 (1H, *t*, *J* = 5.5 Hz), 3.87 (*s*, 3H, COOMe), 3.65 (*s*, 3H, OMe), 2.50–2.20 (*m*, 4H), 1.47 (3H, *d*, *J* = 6.9 Hz, CH_3_).^13^C NMR (50 MHz, CDCl_3_) δ 193.4 (C, C=O), 169.4(C, C=O), 164.1 (C, C_*ipso*_), 160.6 (C, C_*ipso*_), 139.2(C, C_*ipso*_), 127.5 (CH × 2, Ar), 125.0 (CH × 2, Ar), 122.3 (CH, Ar), 122.1 (CH × 2, Ar), 110.4 (CH × 2, Ar), 51.9 (CH_3_, COOMe), 50.1 (CH), 48.1 (CH_3_, OMe), 45.3 (CH), 27.6 (CH_2_), 23.3 (CH_2_), 18.4 (CH_3_). HRMS (EI): C_22_H_26_NO_5_ requires (*M* + H)^+^, 384.1803, found 384.1805.

## Refinement   

Crystal data, data collection and structure refinement details are summarized in Table 2 ▸. The hydrogen atoms were positioned geometrically, with C–H distances constrained to 0.93 Å (aromatic CH), 0.97 Å (methyl­ene CH_2_), 0.98 methyne CH) and N—H = 0.86 Å (amine), and refined using a riding model with *U*_iso_(H) = 1.2 or 1.5*U*_eq_(C,N).

## Supplementary Material

Crystal structure: contains datablock(s) global, I. DOI: 10.1107/S2056989017003607/hb7652sup1.cif

Structure factors: contains datablock(s) I. DOI: 10.1107/S2056989017003607/hb7652Isup2.hkl

Supporting information file. DOI: 10.1107/S2056989017003607/hb7652Isup3.cml

CCDC reference: 1524270

Additional supporting information:  crystallographic information; 3D view; checkCIF report

## Figures and Tables

**Table 1:** Hydrogen-bond geometry (Å, °)

*D*—H⋯*A*	*D*—H	H⋯*A*	*D*⋯*A*	*D*—H⋯*A*
N1—H1⋯O3^i^	0.86	2.02	2.871 (6)	168
C9—H9⋯O3^i^	0.98	2.46	3.277 (7)	141
C20—H20*A*⋯O2^i^	0.97	2.49	3.410 (8)	158
C17—H17⋯O5^ii^	0.93	2.55	3.303 (11)	139

Symmetry codes: (i) 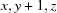; (ii) 
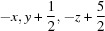
.

**Table 2:** Experimental details

Crystal data	
Chemical formula	C_22_H_25_NO_5_
*M*_r_	383.43
Crystal system, space group	Orthorhombic, *P*2_1_2_1_2_1_
Temperature (K)	298
*a*, *b*, *c* (Å)	23.739 (3), 4.7791 (5), 18.0722 (19)
*V* (Å^3^)	2050.3 (4)
*Z*	4
Radiation type	Cu *K*α
μ (mm^−1^)	0.72
Crystal size (mm)	0.12 × 0.10 × 0.08

Data collection	
Diffractometer	Bruker APEXII CCD area-detector
Absorption correction	Multi-scan (*SADABS*; Bruker, 2006 ▸)
*T*_min_, *T*_max_	0.917, 0.944
No. of measured, independent and observed [*I* > 2σ(*I*)] reflections	9316, 2913, 1854
*R*_int_	0.053
(sin θ/λ)_max_ (Å^−1^)	0.594

Refinement	
*R*[*F* ^2^ > 2σ(*F* ^2^)], *wR*(*F* ^2^), *S*	0.067, 0.163, 1.25
No. of reflections	2913
No. of parameters	256
H-atom treatment	H-atom parameters constrained
Δρ_max_, Δρ_min_ (e Å^−3^)	0.15, −0.19
Absolute structure parameter	0.0 (8)

Computer programs: *APEX2* and *SAINT* (Bruker 2006 ▸), *SHELXS97*, *SHELXL97* and *SHELXTL* (Sheldrick, 2008 ▸) and *Mercury* (Macrae *et al.*, 2008 ▸).

## supplementary crystallographic information

### Crystal data

**Table:**

C_22_H_25_NO_5_	*F*(000) = 816
*M**_r_* = 383.43	*D*_x_ = 1.242 Mg m^−^^3^
Orthorhombic, *P*2_1_2_1_2_1_	Cu *K*α radiation, λ = 1.54178 Å
Hall symbol: P 2ac 2ab	Cell parameters from 3001 reflections
*a* = 23.739 (3) Å	θ = 3.1–60.0°
*b* = 4.7791 (5) Å	µ = 0.72 mm^−^^1^
*c* = 18.0722 (19) Å	*T* = 298 K
*V* = 2050.3 (4) Å^3^	Prismatic, colorless
*Z* = 4	0.12 × 0.10 × 0.08 mm

### Data collection

**Table:**

Bruker APEXII CCD area-detector diffractometer	2913 independent reflections
Radiation source: fine-focus sealed tube	1854 reflections with *I* > 2σ(*I*)
Graphite monochromator	*R*_int_ = 0.053
phi and ω scans	θ_max_ = 66.3°, θ_min_ = 3.1°
Absorption correction: multi-scan (SADABS; Bruker, 2006)	*h* = −26→27
*T*_min_ = 0.917, *T*_max_ = 0.944	*k* = −5→4
9316 measured reflections	*l* = −18→21

### Refinement

**Table:**

Refinement on *F*^2^	Primary atom site location: structure-invariant direct methods
Least-squares matrix: full	Secondary atom site location: difference Fourier map
*R*[*F*^2^ > 2σ(*F*^2^)] = 0.067	Hydrogen site location: inferred from neighbouring sites
*wR*(*F*^2^) = 0.163	H-atom parameters constrained
*S* = 1.25	*w* = 1/[σ^2^(*F*_o_^2^) + (0.0115*P*)^2^ + 2.2937*P*] where *P* = (*F*_o_^2^ + 2*F*_c_^2^)/3
2913 reflections	(Δ/σ)_max_ < 0.001
256 parameters	Δρ_max_ = 0.15 e Å^−^^3^
0 restraints	Δρ_min_ = −0.19 e Å^−^^3^

### Special details

**Table:**

Geometry. All esds (except the esd in the dihedral angle between two l.s. planes) are estimated using the full covariance matrix. The cell esds are taken into account individually in the estimation of esds in distances, angles and torsion angles; correlations between esds in cell parameters are only used when they are defined by crystal symmetry. An approximate (isotropic) treatment of cell esds is used for estimating esds involving l.s. planes.
Refinement. Refinement of F^2^ against ALL reflections. The weighted R-factor wR and goodness of fit S are based on F^2^, conventional R-factors R are based on F, with F set to zero for negative F^2^. The threshold expression of F^2^ > 2sigma(F^2^) is used only for calculating R-factors(gt) etc. and is not relevant to the choice of reflections for refinement. R-factors based on F^2^ are statistically about twice as large as those based on F, and R- factors based on ALL data will be even larger.

### Fractional atomic coordinates and isotropic or equivalent isotropic displacement parameters (Å^2^)

**Table:**

	*x*	*y*	*z*	*U*_iso_*/*U*_eq_	
O1	0.14092 (19)	0.5853 (12)	0.7501 (3)	0.0968 (16)	
O2	−0.07814 (19)	0.4116 (10)	0.9431 (2)	0.0873 (15)	
O3	0.0203 (2)	0.3173 (8)	1.0773 (3)	0.0954 (17)	
O4	−0.2138 (2)	1.1081 (15)	1.0782 (4)	0.135 (2)	
O5	−0.1868 (3)	0.7840 (19)	1.1507 (4)	0.172 (4)	
N1	0.0500 (2)	0.7476 (10)	1.1092 (3)	0.0660 (15)	
H1	0.0457	0.9243	1.1022	0.079*	
C1	0.0970 (3)	0.5689 (16)	0.7980 (4)	0.0737 (19)	
C2	0.0521 (3)	0.3913 (14)	0.7891 (4)	0.077 (2)	
H2	0.0503	0.2712	0.7487	0.093*	
C3	0.0090 (3)	0.3946 (15)	0.8420 (4)	0.0721 (19)	
H3	−0.0216	0.2754	0.8359	0.087*	
C4	0.0105 (3)	0.5687 (13)	0.9029 (4)	0.0620 (16)	
C5	0.0563 (3)	0.7466 (14)	0.9097 (4)	0.0742 (19)	
H5	0.0584	0.8673	0.9500	0.089*	
C6	0.0987 (3)	0.7474 (16)	0.8579 (4)	0.081 (2)	
H6	0.1288	0.8699	0.8632	0.098*	
C7	0.1414 (3)	0.3946 (19)	0.6885 (5)	0.116 (3)	
H7A	0.1399	0.2057	0.7065	0.173*	
H7B	0.1754	0.4207	0.6605	0.173*	
H7C	0.1094	0.4302	0.6575	0.173*	
C8	−0.0366 (3)	0.5519 (12)	0.9563 (4)	0.0622 (16)	
C9	−0.0306 (2)	0.7072 (11)	1.0286 (3)	0.0621 (17)	
H9	−0.0205	0.9019	1.0180	0.075*	
C10	0.0160 (3)	0.5753 (13)	1.0744 (4)	0.0628 (16)	
C11	0.0948 (3)	0.6560 (14)	1.1592 (4)	0.077 (2)	
H11	0.1005	0.4553	1.1508	0.093*	
C12	0.1492 (3)	0.8035 (14)	1.1383 (4)	0.0658 (18)	
C13	0.1712 (4)	0.7725 (19)	1.0682 (5)	0.106 (3)	
H13	0.1523	0.6623	1.0339	0.127*	
C14	0.2205 (4)	0.901 (3)	1.0482 (5)	0.128 (4)	
H14	0.2350	0.8761	1.0009	0.154*	
C15	0.2483 (4)	1.065 (2)	1.0980 (7)	0.120 (3)	
H15	0.2817	1.1533	1.0845	0.144*	
C16	0.2273 (4)	1.100 (2)	1.1666 (6)	0.118 (3)	
H16	0.2465	1.2103	1.2006	0.142*	
C17	0.1779 (3)	0.9729 (15)	1.1865 (4)	0.085 (2)	
H17	0.1635	1.0022	1.2337	0.102*	
C18	0.0765 (3)	0.692 (2)	1.2385 (4)	0.117 (3)	
H18A	0.0712	0.8878	1.2487	0.176*	
H18B	0.1049	0.6181	1.2708	0.176*	
H18C	0.0417	0.5946	1.2464	0.176*	
C19	−0.0847 (2)	0.7043 (12)	1.0759 (3)	0.0672 (17)	
H19A	−0.0969	0.5120	1.0821	0.081*	
H19B	−0.0760	0.7781	1.1245	0.081*	
C20	−0.1329 (2)	0.8718 (15)	1.0434 (4)	0.078 (2)	
H20A	−0.1190	1.0532	1.0277	0.093*	
H20B	−0.1473	0.7757	1.0000	0.093*	
C21	−0.1799 (3)	0.9125 (19)	1.0976 (5)	0.082 (2)	
C22	−0.2616 (3)	1.157 (2)	1.1275 (6)	0.162 (5)	
H22A	−0.2773	0.9809	1.1425	0.243*	
H22B	−0.2898	1.2642	1.1020	0.243*	
H22C	−0.2491	1.2583	1.1703	0.243*	

### Atomic displacement parameters (Å^2^)

**Table:**

	*U*^11^	*U*^22^	*U*^33^	*U*^12^	*U*^13^	*U*^23^
O1	0.084 (3)	0.115 (4)	0.091 (4)	−0.005 (3)	0.004 (3)	−0.005 (4)
O2	0.099 (3)	0.090 (3)	0.072 (3)	−0.043 (3)	−0.008 (3)	−0.009 (3)
O3	0.135 (4)	0.031 (2)	0.120 (5)	0.001 (3)	−0.050 (4)	0.001 (3)
O4	0.108 (4)	0.149 (6)	0.149 (6)	0.035 (4)	0.045 (4)	0.051 (5)
O5	0.159 (6)	0.218 (8)	0.139 (6)	0.052 (6)	0.054 (5)	0.093 (6)
N1	0.087 (3)	0.034 (3)	0.078 (4)	0.003 (3)	−0.032 (3)	0.002 (3)
C1	0.074 (4)	0.068 (4)	0.079 (5)	0.007 (4)	−0.009 (4)	0.008 (5)
C2	0.089 (5)	0.064 (5)	0.078 (5)	−0.001 (4)	−0.008 (4)	−0.010 (4)
C3	0.080 (4)	0.063 (4)	0.073 (5)	−0.014 (4)	−0.008 (4)	−0.002 (4)
C4	0.072 (4)	0.047 (3)	0.067 (4)	−0.003 (3)	−0.013 (3)	0.003 (4)
C5	0.084 (4)	0.063 (4)	0.076 (5)	−0.013 (4)	−0.010 (4)	−0.007 (4)
C6	0.078 (4)	0.075 (5)	0.091 (6)	−0.019 (4)	−0.009 (4)	−0.004 (5)
C7	0.121 (7)	0.126 (7)	0.100 (7)	0.008 (6)	0.018 (5)	−0.032 (7)
C8	0.083 (4)	0.042 (3)	0.062 (4)	−0.012 (3)	−0.017 (4)	0.000 (3)
C9	0.077 (4)	0.036 (3)	0.074 (5)	−0.007 (3)	−0.018 (3)	0.002 (3)
C10	0.082 (4)	0.044 (3)	0.063 (4)	−0.001 (4)	−0.021 (3)	−0.001 (4)
C11	0.094 (5)	0.055 (4)	0.082 (5)	0.005 (4)	−0.037 (4)	0.006 (4)
C12	0.074 (4)	0.054 (4)	0.070 (5)	0.012 (3)	−0.018 (4)	−0.003 (4)
C13	0.104 (6)	0.126 (7)	0.088 (7)	0.009 (6)	−0.010 (5)	−0.034 (6)
C14	0.104 (7)	0.195 (12)	0.085 (7)	0.025 (7)	0.013 (5)	−0.009 (8)
C15	0.090 (6)	0.139 (9)	0.132 (10)	0.002 (6)	0.003 (7)	0.030 (9)
C16	0.097 (6)	0.128 (8)	0.128 (9)	−0.033 (6)	−0.005 (6)	−0.018 (8)
C17	0.092 (5)	0.086 (5)	0.078 (6)	−0.005 (4)	0.000 (4)	−0.013 (5)
C18	0.105 (5)	0.184 (10)	0.063 (5)	−0.036 (6)	−0.020 (4)	0.038 (7)
C19	0.088 (4)	0.054 (4)	0.059 (4)	−0.006 (3)	−0.009 (4)	0.007 (4)
C20	0.077 (4)	0.078 (5)	0.078 (5)	−0.009 (4)	−0.006 (4)	0.016 (4)
C21	0.083 (5)	0.088 (5)	0.075 (6)	−0.005 (5)	−0.001 (4)	0.014 (5)
C22	0.107 (6)	0.185 (11)	0.195 (11)	0.033 (7)	0.073 (7)	0.023 (9)

### Geometric parameters (Å, º)

**Table:**

O1—C1	1.357 (8)	C9—H9	0.9800
O1—C7	1.439 (8)	C11—C18	1.508 (9)
O2—C8	1.215 (6)	C11—C12	1.518 (9)
O3—C10	1.238 (6)	C11—H11	0.9800
O4—C21	1.282 (9)	C12—C17	1.371 (9)
O4—C22	1.461 (8)	C12—C13	1.379 (10)
O5—C21	1.151 (8)	C13—C14	1.369 (11)
N1—C10	1.314 (7)	C13—H13	0.9300
N1—C11	1.461 (7)	C14—C15	1.364 (12)
N1—H1	0.8600	C14—H14	0.9300
C1—C2	1.373 (9)	C15—C16	1.346 (11)
C1—C6	1.378 (9)	C15—H15	0.9300
C2—C3	1.398 (8)	C16—C17	1.369 (10)
C2—H2	0.9300	C16—H16	0.9300
C3—C4	1.380 (8)	C17—H17	0.9300
C3—H3	0.9300	C18—H18A	0.9600
C4—C5	1.386 (8)	C18—H18B	0.9600
C4—C8	1.481 (8)	C18—H18C	0.9600
C5—C6	1.374 (9)	C19—C20	1.515 (8)
C5—H5	0.9300	C19—H19A	0.9700
C6—H6	0.9300	C19—H19B	0.9700
C7—H7A	0.9600	C20—C21	1.498 (9)
C7—H7B	0.9600	C20—H20A	0.9700
C7—H7C	0.9600	C20—H20B	0.9700
C8—C9	1.509 (8)	C22—H22A	0.9600
C9—C10	1.519 (8)	C22—H22B	0.9600
C9—C19	1.542 (8)	C22—H22C	0.9600
			
C1—O1—C7	117.6 (6)	C12—C11—H11	107.4
C21—O4—C22	115.9 (7)	C17—C12—C13	117.3 (7)
C10—N1—C11	123.7 (5)	C17—C12—C11	122.6 (7)
C10—N1—H1	118.1	C13—C12—C11	120.1 (7)
C11—N1—H1	118.1	C14—C13—C12	121.3 (8)
O1—C1—C2	123.9 (7)	C14—C13—H13	119.4
O1—C1—C6	116.3 (7)	C12—C13—H13	119.4
C2—C1—C6	119.8 (7)	C15—C14—C13	119.8 (9)
C1—C2—C3	118.7 (7)	C15—C14—H14	120.1
C1—C2—H2	120.6	C13—C14—H14	120.1
C3—C2—H2	120.6	C16—C15—C14	120.0 (10)
C4—C3—C2	122.2 (6)	C16—C15—H15	120.0
C4—C3—H3	118.9	C14—C15—H15	120.0
C2—C3—H3	118.9	C15—C16—C17	120.3 (9)
C3—C4—C5	117.5 (6)	C15—C16—H16	119.9
C3—C4—C8	117.9 (6)	C17—C16—H16	119.9
C5—C4—C8	124.6 (6)	C16—C17—C12	121.4 (8)
C6—C5—C4	121.0 (7)	C16—C17—H17	119.3
C6—C5—H5	119.5	C12—C17—H17	119.3
C4—C5—H5	119.5	C11—C18—H18A	109.5
C5—C6—C1	120.8 (7)	C11—C18—H18B	109.5
C5—C6—H6	119.6	H18A—C18—H18B	109.5
C1—C6—H6	119.6	C11—C18—H18C	109.5
O1—C7—H7A	109.5	H18A—C18—H18C	109.5
O1—C7—H7B	109.5	H18B—C18—H18C	109.5
H7A—C7—H7B	109.5	C20—C19—C9	114.2 (5)
O1—C7—H7C	109.5	C20—C19—H19A	108.7
H7A—C7—H7C	109.5	C9—C19—H19A	108.7
H7B—C7—H7C	109.5	C20—C19—H19B	108.7
O2—C8—C4	121.0 (6)	C9—C19—H19B	108.7
O2—C8—C9	121.2 (6)	H19A—C19—H19B	107.6
C4—C8—C9	117.8 (5)	C21—C20—C19	112.2 (6)
C8—C9—C10	109.7 (5)	C21—C20—H20A	109.2
C8—C9—C19	113.3 (5)	C19—C20—H20A	109.2
C10—C9—C19	107.5 (5)	C21—C20—H20B	109.2
C8—C9—H9	108.7	C19—C20—H20B	109.2
C10—C9—H9	108.7	H20A—C20—H20B	107.9
C19—C9—H9	108.7	O5—C21—O4	121.9 (9)
O3—C10—N1	123.6 (6)	O5—C21—C20	125.6 (9)
O3—C10—C9	119.7 (6)	O4—C21—C20	112.5 (7)
N1—C10—C9	116.7 (5)	O4—C22—H22A	109.5
N1—C11—C18	110.0 (6)	O4—C22—H22B	109.5
N1—C11—C12	109.0 (5)	H22A—C22—H22B	109.5
C18—C11—C12	115.3 (6)	O4—C22—H22C	109.5
N1—C11—H11	107.4	H22A—C22—H22C	109.5
C18—C11—H11	107.4	H22B—C22—H22C	109.5

### Hydrogen-bond geometry (Å, º)

**Table:**

*D*—H···*A*	*D*—H	H···*A*	*D*···*A*	*D*—H···*A*
N1—H1···O3^i^	0.86	2.02	2.871 (6)	168
C9—H9···O3^i^	0.98	2.46	3.277 (7)	141
C20—H20*A*···O2^i^	0.97	2.49	3.410 (8)	158
C17—H17···O5^ii^	0.93	2.55	3.303 (11)	139

Symmetry codes: (i) *x*, *y*+1, *z*; (ii) −*x*, *y*+1/2, −*z*+5/2.
